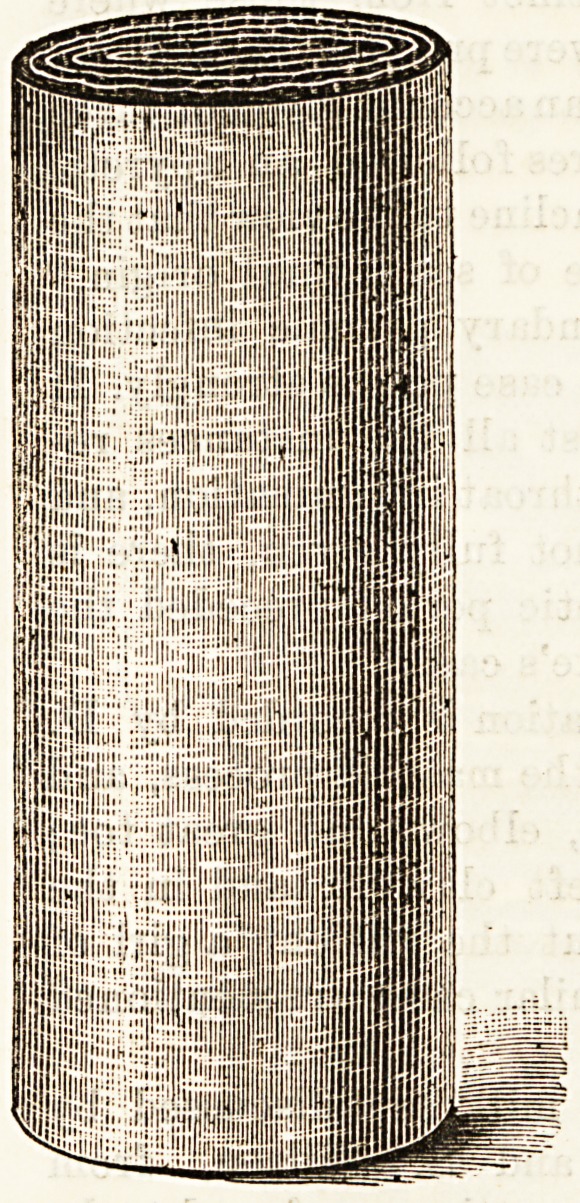# New Appliances and Things Medical

**Published:** 1894-07-14

**Authors:** 


### NEW APPLIANCES AND THINGS MEDICAL.

[All preparations, appliances, novelties, &c., of which a notice is desired, should be sent for the Editor, to care of The Manager, 428, Strand, London, W.C.]

### KINGZETT'S PATENT "SULPHUGALOIS."

(The Sanitas Co. (Limited), Three Colt Lane, London, E.)

We have received a sample box of these most useful novelties, and cannot speak too highly of them as a substitute for the usual method of the burning of solid sulphur, or the more dangerous expedient of setting light to that highly inflammatory substance, carbon bisulphide. The sulphugalois consists of a combustible fabric coated with sulphur and rolled up in the form of a bandage, and in each box there is a holder or cage in which they may be placed while burning They are intended for fumigating bird cages, rabbit hutches, dog kennels, stables, &c.; and where sulphur is employed in the disinfecting of sick rooms, sulphugalois will be found the greatest boon, and in this respect we call the attention of the authorities of public institutions to this useful innovation.

### CADBURY'S COCOA ESSENCE.

(Cadbury and Co., 2, Rood Lane, E.C.)

We have received samples of the above cocoa essence, as well as of chocolate and chocolate biscuits by the same firm. At the present day the market is flooded to such an extent by indifferent and impure brands, and the public are so apt to mistake enterprise in advertisement for excellence of quality, that we cannot too strongly insist on the importance of private individuals and public institutions recognising at once those brands which are pure and those which are contaminated by added and foreign matter. Cadbury's cocoa is absolutely free from the usual contamination of many similar articles, namely, sugar, starch, and potassium sulphate. By the free addition of these substances foreign and other manufacturers are not only able to undersell the producers of the pure article, but their goods are often possessed of a fictitious appearance of strength which entirely deceives the public. The latter, we are aware, dislikes being undeceived in such matters, but we feel it our duty to do so, both for the sake of the honest manufacturer and the long-suffering consumer.

## Figures and Tables

**Figure f1:**